# More powerful significant testing for time course gene expression data using functional principal component analysis approaches

**DOI:** 10.1186/1471-2105-14-6

**Published:** 2013-01-16

**Authors:** Shuang Wu, Hulin Wu

**Affiliations:** 1 Department of Biostatistics and Computational Biology, University of Rochester, 601 Elmwood Avenue, Rochester, NY, 14642, USA

**Keywords:** Differentially expressed genes, Functional data analysis, Multiple group test, One group test, Time course gene expression, Yeast cell cycle

## Abstract

**Background:**

One of the fundamental problems in time course gene expression data analysis is to identify genes associated with a biological process or a particular stimulus of interest, like a treatment or virus infection. Most of the existing methods for this problem are designed for data with longitudinal replicates. But in reality, many time course gene experiments have no replicates or only have a small number of independent replicates.

**Results:**

We focus on the case without replicates and propose a new method for identifying differentially expressed genes by incorporating the functional principal component analysis (FPCA) into a hypothesis testing framework. The data-driven eigenfunctions allow a flexible and parsimonious representation of time course gene expression trajectories, leaving more degrees of freedom for the inference compared to that using a prespecified basis. Moreover, the information of all genes is borrowed for individual gene inferences.

**Conclusion:**

The proposed approach turns out to be more powerful in identifying time course differentially expressed genes compared to the existing methods. The improved performance is demonstrated through simulation studies and a real data application to the *Saccharomyces cerevisiae* cell cycle data.

## Background

Time course microarray and RNA-seq experiments are increasingly used to study biological phenomena that evolve in a temporal fashion. Unlike the static experiment which captures only a snapshot of the gene expression, the time course experiment monitors the gene expression levels over several time points in a biological process, allowing investigators to study dynamic behaviors of the genes. One goal of such experiments is to identify genes associated with a biological process of interest or a particular stimulus, like a therapeutic treatment or virus infection. The differentially expressed genes can be defined as genes with expressions changed significantly with respect to time or across multiple conditions.

The time course gene expression data typically exhibit features such as high dimensionality, short time course, few or no replicates, missing values, large measurement errors, correlations between observations over time, etc. Many of the multivariate techniques for analyzing such data, for example, SAM [1,2], ANOVA [3,4] and empirical Bayes [5], suffer from serious limitations when facing missing data or non-uniformly sampled data. They also fail to account for correlations between measurements from the same gene and do not facilitate the removal of noise from the measured data. In addition, the timing information of when the measurements are taken is not utilized and the inherent temporal structure of the time course data is ignored. A hidden Markov model has been proposed by [6] and [7], where the observed gene profiles are considered to be influenced by an underlying Markov process. The computation of this model involves a large number of parameters, which can be difficult if there are no replicated data, and it can not be applied if the observation time points are distinct for different experimental groups.

Functional data analysis approaches view the expression profile of each gene as a smooth function of time, and the time course measurements are collected as discrete observations from the function that are contaminated by noisy signals [8,9]. A key step is to create an estimate of the gene expression curve from the noisy functional data and this usually involves representing the expression curve as a linear combination of a finite number of basis functions, such as polynomials [10] and B-splines [11-13]. Another popular approach for representing functional curves is the Functional Principal Component Analysis (FPCA) [14,15]. In the FPCA model, the basis functions are estimated from the observed data and the data-adaptive basis has the favorable property to flexibly characterize the major modes of variation in the data. So fewer number of basis functions are needed to capture the shape of gene expression patterns than that using the pre-specified basis.

Many of the existing functional methods are designed for time course data with longitudinal replicates. [13] used a functional hierarchical model and empirical Bayes techniques to determine differentially expressed genes, but the estimates of the model parameters can be very unstable if the number of replicates is small. [16] proposed a functional ANOVA model and [14] adopted the FPCA model for identifying differentially expressed genes across two conditions. In both methods, the model is fitted for one gene at a time to estimate the gene-specific group mean and the covariance structure, which is computationally intensive and may result in overfitted models for a small sample size. [15] adopted a similar approach as [13] to impose a mixture distribution on the gene-specific variation and employed an indicator to reflect whether a gene is differentially expressed. All of these methods require longitudinal replicates in data and only apply to two group comparisons.

Time course data with longitudinal replicates are costly and rather rare in reality. Many of the published time course data have no replicates or only a small number of independent replicates [17-19]. The EDGE method proposed by [12] is a comprehensive approach that is suitable for data with or without replicate, and for both single group and multiple group tests. It represented gene expression trajectories using natural cubic splines and then compared the goodness-of-fit of the model under the null hypothesis to that under the alternative hypothesis. The null distribution of test statistics was approximated by bootstrap. [20] recently extended this method under a permutation-based multiple testing framework. Specifically for time course data without replicate, [21] developed a statistics to measure the signal-to-noise ratio by comparing the energies of the smoothing convolution and differential convolution of the expression profiles. A common problem to these existing methods is that the test statistics are constructed for each gene separately. So they may not be powerful enough to identify differentially expressed genes for short time series data and data without replicates.

In this work, we propose a unified approach to model the gene profiles using the techniques of FPCA, and to identify differentially expressed genes in both single group test and multiple group test. Our methodology is motivated by the gene expression data without replicate, so we will focus on this case in this paper, although our method can also be easily adapted to accommodate data with replicates. We argue that our method can improve the power in identifying differentially expressed genes compared to existing methods. First, using the eigen-basis enables a parsimonious modeling of the gene expression curves, so we have more degrees of freedom for the inference than that using a pre-specified basis. Moreover, we propose to estimate the expression curve of a gene by borrowing strength across all the genes, which leads to a more powerful inference than that using the information of one gene only.

The remainder of the paper is organized as follows. We first describe the FPCA model for representing the gene expression curves and then elaborate a hypothesis testing method based on random permutations to identify differentially expressed genes in both single group and multiple group scenarios. The proposed method is compared with several existing methods via the analysis of the *Saccharomyces cerevisiae* cell cycle data from [17] and simulation studies. Lastly, we summarize the proposed method and discuss possible extensions.

## Methods

### FPCA for time course gene expression data

We adopt the point of view in functional data analysis to consider the time course gene expression curves as a sample of random functions. We further assume that the expression profile *X*(*t*) of a single gene is a smooth function in the time interval [*a*,*b*], with mean function *μ*(*t*) = E (*X*(*t*)) and covariance function *G*(*s*,*t*) = cov (*X*(*s*), *X*(*t*)). Under mild conditions, we can assume that *X* possesses Karhunen-Loéve representation [22] with representation

(1) $$X(t)=\mu (t)+\overset{\infty }{\underset{l=1}{\sum }}\xi_{l}\phi_{l}(t),$$

where *ϕ*_*l*_ are sequences of orthonormal eigenfunctions with non-increasing eigenvalues *λ*_*l*_, satisfying ∑*lλ*_*l*_<*∞* and *G*(*s*,*t*)= ∑*l*=1*λ*_*l*_*ϕ*_*l*_(*s*)*ϕ*_*l*_(*t*). These eigenfunctions reflect the direction of major shape deviations from the mean function and the random coefficient *ξ*_*l*_, often referred to as the functional principal component (FPC) score, indicates how much a gene deviates from the mean function in the direction of *ϕ*_*l*_.

The observed gene expression data are assumed to be discrete observations from the true expression curves which are further disrupted by noisy signals. For convenience of presentation, we assume that there are no replicates. The model for the observed data can be written as

(2) $$Y_{\text{ijk}}\;=\;X_{\text{ij}}(t_{\text{ijk}})\;+\;\varepsilon_{\text{ijk}},\;i\;=\;1,\ldots ,n,\;j\;=\;1,\ldots ,J,\;k=1,\ldots ,K_{\text{ij}},$$

where *n* is the number of genes, *J* is the number of experimental groups, and *K*_*ij*_ is the number of sampling time points for gene *i* in the *j*-th experimental group. The noises *ε*_*ijk*_ are assumed to be i.i.d. random variables with mean 0 and variance *σ*^2^. In this paper, we adopt the PACE method – principal component analysis through conditional expectation proposed by [23] to estimate ${\hat{X}}_{\text{ij}}(t)$ from the observed noisy data. This approach borrows information across all the genes to predict individual expression curves and is more efficient than the gene-specific smoothing method when dealing with thousands of genes simultaneously.

**Figure 1:**
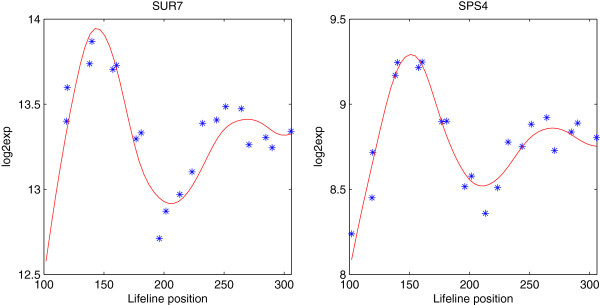
**Time course measurements (star) and the estimated expression curves (solid) for genes SUR7 and SPS4 in the yeast cell cycle data, plotted in *log2 *scale.**

In real data analysis, some genes may share similar expression patterns but with dramatically different magnitude levels, for example, genes SUR7 and SPS4 in the yeast cell cycle data shown in Figure 1. Using model (1), this magnitude difference will be modeled as the random variation around the population mean *μ*(*t*), which is not efficient, as the magnitude difference may obscure some other interesting variations. So we first subtract the mean expression of each gene from the observed data and apply FPCA to the centered data. The empirical covariances are calculated from the aggregated data of all genes as

(3) $$C_{j}(t_{k},t_{l})=\frac{1}{n}\overset{n}{\underset{i=1}{\sum }}(Y_{\text{ijk}}-{\hat{\mu }}_{\text{ij}})(Y_{\text{ijl}}-{\hat{\mu }}_{\text{ij}}),$$

where ${\hat{\mu }}_{\text{ij}}=\overset{K_{\text{ij}}}{\underset{k=1}{\sum }}Y_{\text{ijk}}/K_{\text{ij}}$. We then obtain the estimate of the covariance function *G*(*s*,*t*) by applying two-dimensional local linear smoothers to (3). Note that the diagonal elements *C*_*j*_(*t*_*k*_,*t*_*k*_) should not be used in estimating *G*(*s*,*t*) because they are contaminated by the noise signal. When replicates are present, we can apply the above procedure to the averaged expression data ${\bar{Y}}_{\text{ijk}}=\frac{1}{M_{j}}\underset{m}{\sum }Y_{\text{ijkm}}$, where *M*_*j*_ is the number of replicates for group *j*.

The eigenfunctions and eigenvalues are estimated by numerical spectral decomposition of Ĝ(s,t) for a suitably discretized version. The FPC scores can be estimated by approximating the integral $\xi_{\text{ijl}}=\int_{a}^{b}(X_{\text{ij}}(t)-\mu (t))\phi_{l}(t)\text{dt}$ if the observed data are dense. Alternative shrinkage estimators for sparse and irregular data are described in [23]. Analogous to the principal component analysis in multivariate analysis, the total variation in the data can be largely explained by the first few functional principal components. So we use the first *L* eigenfunctions to approximate ${\hat{X}}_{\text{ij}}$ and *L* can be chosen by the information criteria such as AIC, BIC, or by scree plot or fraction of variation explained (FVE), similarly as in the PCA in multivariate analysis. More details for the estimation procedure can be found in [23]. With the estimates of all the model components in hand, we can now represent the individual gene expression trajectories as

(4) $${\hat{X}}_{\text{ij}}(t)={\hat{\mu }}_{\text{ij}}+\overset{L_{j}}{\underset{l=1}{\sum }}{\hat{\xi }}_{\text{ijl}}{\hat{\phi }}_{\text{jl}}(t).$$

### Identifying differentially expressed genes

#### One group case

In the case of a single experimental group (*J*=1), we are often interested in discovering genes whose expression profiles are time-dependent. We want to test whether the expression curve is constant for gene *i*, *i*=1,…,*n*, i.e.

(5) $$\;H_{i0}:\;X_{i}(t)=\mu_{i},\;\mathrm{v.s.}\;H_{i1}:\;X_{i}(t)\neq \mu_{i},\;\;\;\text{for all}\;\;t\in [a,b].$$

Under *H*_*i*0_, the constant is estimated as the sample mean ${\hat{X}}_{i}^{0}(t)={\hat{\mu }}_{i}=\overset{K_{i}}{\underset{k=1}{\sum }}Y_{\text{ik}}/K_{i}$, and under *H*_*i*1_, the curve estimate ${\hat{X}}_{i}^{1}(t)$ is obtained as (4). The test statistic is a modified *F*-statistic, which compares the goodness-of-fit of the null model to the alternative model:

(6) $$F_{i}=\frac{{\text{RSS}}_{i}^{0}-{\text{RSS}}_{i}^{1}}{{\text{RSS}}_{i}^{1}+\delta },$$

where ${\text{RSS}}_{i}^{0}$ and ${\text{RSS}}_{i}^{1}$ are the residual sum of squares under the null and the alternative models, respectively. In a typical time course gene expression data, the number of time points is the same for all genes within one experimental group. Since we use the same number of eigen-basis functions to approximate the gene expression curves, dividing the numerator and denominator of (6) by the corresponding degrees of freedom will not change the ordering of the test statistics. In case that there are missing values in the data, one could adjust (6) correspondingly.

This statistic can also be viewed as the signal-to-noise ratio of each gene. For genes with a low signal level, variance in *F*_*i*_ can be high because of small values of ${\text{RSS}}_{i}^{1}$. The small constant *δ* in the denominator can help stabilize the variance of *F*_*i*_. A similar idea has been adopted in [1]. In this work, we set $\delta ={\hat{\sigma }}^{2}$, the estimated variance of the noisy signal in (2). Since cov(*Y*_*ik*_,*Y*_*il*_)=cov(*X*_*i*_(*t*_*ik*_),*X*_*i*_(*t*_*il*_))+*σ*^2^*δ*_*kl*_, where *δ*_*kl*_=1 if *k*=*l* and 0 otherwise, we can estimate *σ*^2^ by smoothing (3) with and without the diagonals *C*_*j*_(*t*_*k*_,*t*_*k*_). Specifically, *σ*^2^ can be estimated by the averaged difference between the local linear smoother along the diagonal of the raw covariance and a local quadratic smoother along the direction perpendicular to the diagonal. See [23] for more details.

There is a sizable literature on the asymptotic distribution of *F*_*i*_, for example [24]. However, such methods are generally not applicable to time course gene expression data, as the number of measurements for each gene is usually very small. In order to generate the null distribution of (*F*_1_,…,*F*_*n*_), we propose using a permutation test. Each permutation sample is generated by randomly matching the expression measurements of *n* genes with their sampling times. If there are replicates available, the expression measurements at the same time point are permuted as a group. For example, let *Y*_*ikm*_ be the *m*-th measurements for gene *i* at time *t*_*k*_, *m*=1,…,*M*. The permutation samples are obtained by randomly shuffling the time index *k*. To facilitate the computing efficiency, we use the eigenfunctions obtained from the observed data for the permutation samples.

With the *F*-statistics of the permutation samples computed from (6), the *p*-value for gene *i* can be defined as

(7) $$p_{i}=\overset{B}{\underset{b=1}{\sum }}\frac{I\{F_{i}^{\left(b\right)}\geq F_{i}\}}{B},$$

where *B* is the number of permutation samples, *I*(·) is an indicator function and $F_{i}^{\left(b\right)}$ is the statistic computed from the *b*-th permutation. [12] proposed another definition of the *p*-value by considering the permutation statistics from all genes:

(8) $$p_{i}=\overset{B}{\underset{b=1}{\sum }}\overset{n}{\underset{j=1}{\sum }}\frac{I\{F_{j}^{\left(b\right)}\geq F_{i}\}}{n\cdot B}.$$

This definition has the advantage that the ordering of the test statistics is preserved in the ordering of the *p*-values. We also find in our simulations that (8) leads to fewer false positives than (7). Therefore, we adopt (8) in the real data application.

When we apply the proposed procedure to identify differentially expressed genes, it is necessary to consider the multiple testing adjustment because *n* hypotheses are tested simultaneously and the number of genes *n* is usually very large. A commonly used strategy is to control the false discovery rate (FDR), which has been studied in various literature, including [2,25] and [26]. We adopt the one proposed in [25] since it is easy to compute and widely accepted.

#### Multiple group case

In the multiple group setting, we want to identify genes with different expression profiles in different experimental groups. The hypothesis for gene *i* can be written as

(9) $$\begin{array}{l} H_{i0}:\;X_{i1}(t)\;=\;\ldots \;=\;X_{\text{iJ}}(t),\;\mathrm{v.s.}\;H_{i1}:\;X_{\text{ij}}(t)\;\neq \;X_{ij^{\prime }}(t),\;j\neq j^{\prime },\\ \text{for all}\;\;t\in [a,b]. \end{array}$$

The estimates ${\hat{X}}_{\text{ij}}^{1}(t)$ under *H*_*i*1_ are obtained as (4) by using the data from group *j* only. Under *H*_*i*0_, the group-free estimates ${\hat{X}}_{i}^{0}(t)$ can be obtained using the pooled data from *J* groups. The residual sum of squares under the null and the alternative models are calculated as ${\text{RSS}}_{i}^{0}=\overset{J}{\underset{j=1}{\sum }}\overset{K_{j}}{\underset{k=1}{\sum }}(Y_{\text{ijk}}-{\hat{X}}_{i}^{0})$ and ${\text{RSS}}_{i}^{1}=\overset{J}{\underset{j=1}{\sum }}\overset{K_{j}}{\underset{k=1}{\sum }}(Y_{\text{ijk}}-{\hat{X}}_{\text{ij}}^{1})$, respectively, and the *F*-statistics are computed as (6), where $\delta =\max(\overset{2}{\underset{j}{\hat{\sigma }}})$ with ${\hat{\sigma }}_{j}^{2}$ being the estimated variance of the noisy signal for the *j*-th group.

We again use a permutation test to obtain the null distribution of (*F*_1_,…,*F*_*n*_). Without loss of generality, we assume that the measurement times are the same among the *J* groups and at least one observation is available at each time point for each group. A permutation sample is generated by permuting the pooled gene expression data at each time point, i.e., the data {*Y*_*ijk*_,1≤*j*≤*J*} are randomly partitioned into *J* groups. If replicates are available, i.e., {*Y*_*ijkm*_,1≤*m*≤*M*_*j*_,1≤*j*≤*J*}, the measurements are randomly partitioned into *J* groups of sizes *M*_1_,…,*M*_*J*_. The calculation of *p*-values and the multiple testing adjustment are the same as described in the previous subsection. This approach can also be extended to situations where the measurement time points vary across different experimental groups. In this case, we can divide the time interval [*a*,*b*] into small bins so that at least one observation falls into each bin for each group. We then permute the gene expression data within each bin instead of at each time point. This extended approach is further illustrated with the yeast cell cycle data in the next section.

## Results and discussion

### Yeast cell cycle data

In this section, we applied the proposed method to *Saccharomyces cerevisiae* cell cycle gene expression data reported in [17]. The dataset includes the gene expression measurements of *n*=10928 probe sets for both wild type and cyclin mutant cells at 30 different time points. In the following presentation, we refer to the probe sets as genes. The clock time points are aligned to the corresponding lifeline positions, covering about two cell cycles in the wild type and about 1.5 cell cycles in the cyclin mutant. [17] found that 1275 genes were transcribed periodically and 835 of these periodic genes showed changes in expression behaviors in the cyclin mutant. In the following analysis, we use the numerical lifeline positions as indicators for time. The measurement times are irregular for both the wild type and the cyclin mutant, and in addition the time points are different among these two groups. We take log2 transformation of the original data.

#### Differentially expressed genes in wild type cells

**Figure 2:**
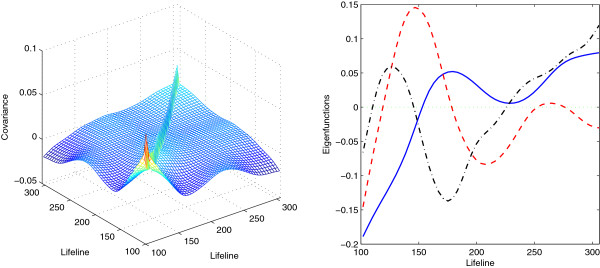
Estimates for the covariance function (left panel) and the first three eigenfunctions (right panel; first-solid, second-dashed, third-dash dotted) for the wild type data with lifeline position greater than 100.

We first apply the single group test to the wild type data to identify differentially expressed genes with non-flat expression profiles. The lifeline positions of the wild type data range from 14 to 305, where 0-100 correspond to the recovery phase from synchrony and 100-305 correspond to two cell cycles. We take the data with lifeline greater than 100, including 21 time points. Applying the FPCA method, we select the first three principal components, accounting for 97.5% of the total variation. Figure 2 displays the estimated covariance function and the first three eigenfunctions. The ridge along the diagonal of the covariance function depicts the variance of the noisy signal. The first eigenfunction (explaining 65.6% of total variation) has an increasing trend with small fluctuations after lifeline 180. Both the second and the third eigenfunctions (explaining 17.7% and 14.2% of total variation, respectively) exhibit clear periodic patterns. The second eigenfunction has two peaks around lifeline 150 and 260, corresponding to the two cell cycles, respectively. The third eigenfunction displays a seemingly sinusoidal wave up to lifeline 200 and increases linearly afterwards.

Using *B*=10,000 permutations, we identified 1180 genes with *FDR*=0.01, in which 750 are shared with the list of 1275 genes identified by [17]. We also find that these 750 shared genes constitute 82% of the 500 top ranked genes in Orlando’s list. The discrepancy between genes identified by [17] and by our method may due to the differences in the selecting criteria. [17] emphasized on the periodic pattern of the gene expressions, while our method aims to maximize the ratio of the variation around the mean expression and the noise level. So genes included in Orlando’s list but missed by our method may have large noises in the observed data. On the other hand, genes identified by our method could have non-periodic patterns, for example, genes with large loadings on the first eigenfunction may exhibit linear trends.

It is also of interest to compare the results from the proposed method with those of the EDGE method proposed by [12] and the permutation-based method by [20]. Since there are no longitudinal replicates available, the methods in [14] and [15] are not applicable. In both EDGE and Sohn’s methods, the gene expression curves are estimated using the gene-specific B-spline smoothing, and the number of spline basis functions is determined as the sufficient size for fitting all the top “eigen-genes”. For this dataset, nine B-spline basis functions are used. EDGE used a bootstrap method for approximating the null distribution of the statistics, while Sohn’s method adopted a similar permutation approach as our method. In order for the results from different methods to be comparable, we also use the FDR procedure proposed by [25] in the EDGE and Sohn’s methods. Table 1 shows the numbers of genes identified by these two methods and their overlaps with genes identified by [17] and our proposed method. We can see that our method identified the most genes and had the highest agreement with the significant gene list in [17].

**Table 1:** Identified gene numbers by the proposed method, EDGE and Sohn’s method for testing non-flat gene expressions in the wild type cells, adjusted at FDR levels of 0.01 and 0.05

	**FDR = 0.01**			**FDR = 0.05**				
	**Genes**	**Overlap**	**Overlap**	**Genes**	**Overlap**	**Overlap**		
	**identified**	**w/Orlando**	**w/proposed**	**identified**	**w/Orlando**	**w/proposed**		
Proposed	1180	750	-	1958	901	-
EDGE	1076	617	809	1705	783	1322
Sohn’s	985	590	767	1636	769	1291

The numbers of genes overlapped with the list by [17] and the proposed method are also included.

**Figure 3:**
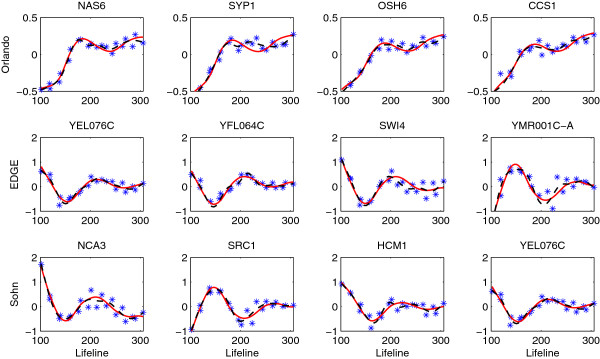
**Genes selected by the proposed method but not by **[17]**(first row), EDGE (second row) and Sohn’s method (third row) for testing non-flat expressions in the wild type data (FDR = 0.01).** The observed expressed values (star) and the estimated expression curves (FPCA-solid, dashed-B splines) are centered.

**Figure 4:**
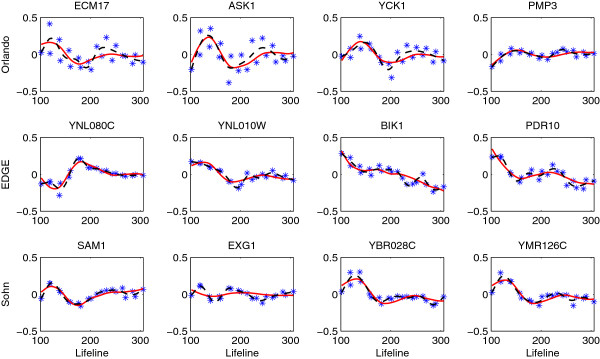
**Genes not selected by the proposed method but by **[17]**(first row), EDGE (second row) and Sohn’s method (third row) for testing non-flat expressions in the wild type data (FDR = 0.01).** The observed expressed values (star) and the estimated expression curves (FPCA-solid, dashed-B splines) are centered.

Figure 3 displays the gene expression profiles chosen as differentially expressed by our method but not by others, while Figure 4 reflects the opposite scenario showing the profiles of genes chosen by other methods only. We can see that genes identified by our method but not by [17] have similar shapes to the first eigenfunction, and our method missed those periodic genes with either large noises or small variations around the mean expression. The genes picked by EDGE or Sohn’s method but not by our method mostly have small variations around the means. Since the *F*-statistic (6) is a signal-to-noise ratio, these genes with small “signals” would still have large statistics due to small noises. In addition, the expression curves estimated by the gene-specific B-spline smoothing tend to be under-smoothed (Figure 4, last two rows), which makes the denominator of the *F*-statistic even smaller. These genes are not identified by our method because we add a small constant in the denominator of (6) to stabilize its variance.

#### Comparing genes in wild type and cyclin mutant cells

We next apply our method to identify genes with different expression patterns in the wild type and cyclin mutant cells. We restrict this analysis to the 1275 periodic genes identified by [17]. Since the maximum lifeline position for the cyclin mutant is 243.8, we consider the gene expressions within interval [100,244], which includes 15 time points for the wild type and 22 time points for the cyclin mutant. The observation times are not equally spaced and are not the same for the two cell types.

**Figure 5:**
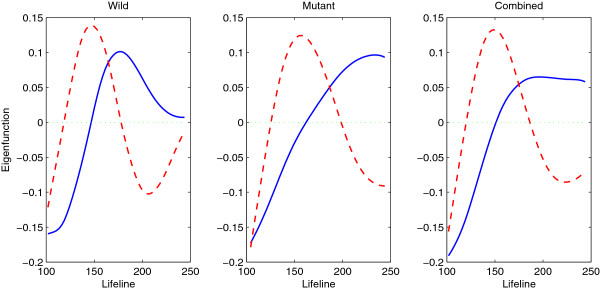
Estimated eigenfunctions (first-solid, second-dashed) for the wild type (left), the cyclin mutant (middle) and the combined data (right).

We apply the FPCA method to the wild type, the cyclin mutant and the combined samples and obtain the estimates of their eigenfunctions. Figure 5 shows the estimates of the first two eigenfunctions, accounting for 94.9% of the total variation for the wild type, 99.6% for the cyclin mutant and 98.9% for the combined data. We can see that the eigenfunctions of different samples have some similarity, but also show some clear differences: The first eigenfunction of the wild type (explaining 69.4% of total variation) increases up to around lifeline 180 and starts to decrease afterwards, while the first eigenfunction of the cyclin mutant (explaining 80.4% of total variation) increases at a slower rate but keeps increasing till the end; the second eigenfunction of the wild type (explaining 25.5% of total variation) has a sinusoidal shape with a peak around lifeline 150 and a trough around lifeline 200, while the second eigenfunction of the cyclin mutant (explaining 19.2% of total variation) peaks a little later and does not exhibit an increase pattern after lifeline 200; the eigenfunctions of the combined samples seem to be the average of the corresponding eigenfunctions of the wild type and the cyclin mutant. [17] found that, although the cyclin mutant cells are devoid of functional Clb-CDK complex and arrest at the G1/S-phase border, a majority of their genes continued to be expressed on schedule, with minor changes in transcript behavior in comparison with wild type cells. This explains the similarity of the eigenfunctions of the wild type and cyclin mutant. In addition, we find that the cell cycle of the cyclin mutant cells seem to be 50% longer than that of the wild type, so within 1.5 cell cycles of the wild type, the cyclin mutant cells only have one cell cycle.

Since the observation times are different for the wild type and the cyclin mutant, we can not use the usual permutation. Instead, we divide the lifeline domain [100,244] into small bins with length 10 each. Within each bin, there are at least one observation from each of the wild type and the cyclin mutant groups (except for [120,130], which includes no observations from either the wild type or the cyclin mutant). The observations are permuted within each bin, using the permutation strategy for data with replicates as described in Section “Multiple group case”.

Using *B*=10,000 permutations, we identified 883 genes for an FDR of 0.01, in which 631 are included in the 835 genes identified by [17] with changed expression patterns in the cyclin mutant. These genes are likely to be directly or indirectly regulated by Clb-CDK, and since Clb-CDK activities are known to be essential for triggering the transcriptional programme, we may not observe any periodic expression patterns in these genes for the cyclin mutant cells. For this data, ten B-spline basis functions are used in EDGE and Sohn’s methods for smoothing the expression data. The numbers of differential genes identified by EDGE and Sohn’s methods are 704 and 522 for *FDR*=0.01, respectively, sharing 451 and 329 genes with those identified by [17] (Table 2).

**Table 2:** Identified gene numbers by the proposed method, EDGE and Sohn’s method for comparing gene expression profiles between the wild type and the cyclin mutant, adjusted at FDR levels of 0.01 and 0.05, respectively

	**FDR = 0.01**			**FDR = 0.05**				
	**Genes**	**Overlap**	**Overlap**	**Genes**	**Overlap**	**Overlap**		
	**identified**	**w/Orlando**	**w/proposed**	**identified**	**w/Orlando**	**w/proposed**		
Proposed	883	631	-	1086	735	-
EDGE	704	524	679	839	600	833
Sohn’s	522	410	516	758	557	755

The numbers of genes overlapped with the list by [17] and the proposed method are also included.

**Figure 6:**
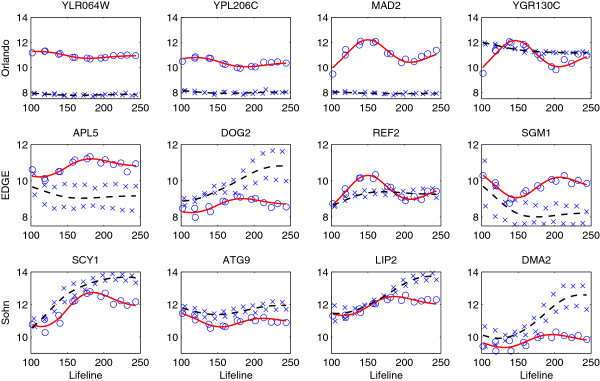
**Genes selected by the proposed method but not by **[17]**(first row), EDGE (second row) and Sohn’s method (third row) for testing changes in expressions between the wild type and the cyclin mutant groups (FDR = 0.01).** The observed expressed values for the wild type (circle) and the cyclin mutant (cross) are displayed and overlaid with the corresponding estimated expression curves (wild-solid, mutant-dashed).

**Figure 7:**
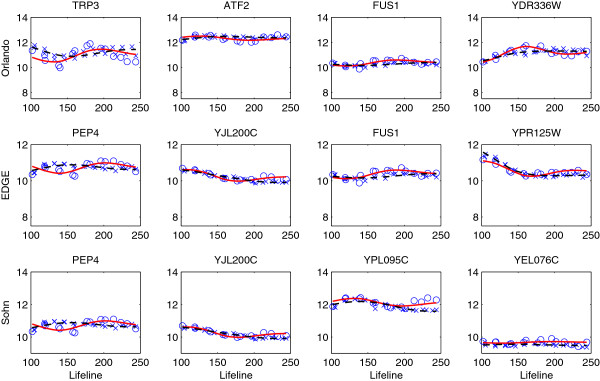
**Genes not selected by the proposed method but by **[17]**(first row), EDGE (second row) and Sohn’s method (third row) for testing changes in expressions between the wild type and the cyclin mutant groups (FDR = 0.01).** The observed expressed values for the wild type (circle) and the cyclin mutant (cross) are displayed and overlaid with the corresponding estimated expression curves (wild-solid, mutant-dashed).

Most of the genes identified by EDGE and Sohn’s methods are also identified by our method, but our method identified some genes that are not picked by the other two methods. This is because the EDGE and Sohn’s methods used a large number of B-spline basis for smoothing the bootstrap/permutation samples, which generally leads to under-smoothed fits. This may inflate the probability of large valued statistics under the null hypothesis, so fewer genes would be identified. In Figure 6, we display 4 randomly picked genes that are selected by our method but not by [17], EDGE or Sohn’s method. [17] seems to miss some genes with vertical shifts in their expression levels. Figure 7 shows genes that are not selected by our method but by others. The y-axis is adjusted to have the same scale of that in Figure 6 for easier comparison. The comparison confirms that the proposed method can detect significant patterns that the other methods fail to identify.

### Simulation studies

Simulation studies are carried out to compare the performance of our method to that of the EDGE method and the permutation-based method by [20]. We consider a single group test of non-flat gene expressions and a two group test of differential gene expressions across groups. In both cases, the data are simulated to mimic the *Saccharomyces cerevisiae* cell cycle data set.

In the single group case, the non-differential genes have model *X*_*i*_(*t*)=0, and the differential genes have $X_{i}(t)=\overset{3}{\underset{l=1}{\sum }}\xi_{\text{il}}\phi_{l}(t)$, where $\phi_{1}(t)=-\sqrt{2/(b_{1}-a_{1})}\cos\left(\right)2\Pi (t-a_{1})/(b_{1}-a_{1})))$, $\phi_{2}(t)=\sqrt{2/(b_{1}-a_{1})}\sin\left(\right)2\Pi (t-a_{1})/$ (*b*_1_−*a*_1_)) and $\phi_{3}(t)=-\sqrt{2/(b_{1}-a_{1})}\cos\left(\right)4\Pi (t-a_{1})/(b_{1}-a_{1})))$, *t*∈[*a*_1_,*b*_1_]. The coefficients *ξ*_*il*_ are i.i.d. normal r.v.’s with mean 0 and variance *λ*_*l*_ with *λ*_1_=4, *λ*_2_=2 and *λ*_3_=1. For each gene, the gene expression profiles are simulated at the same time points as the wild type yeast data, so there are 21 observations in [*a*_1_,*b*_1_]=[100,305]. The noisy signal in the observed data is simulated as i.i.d. normal r.v. with mean 0 and variance *σ*^2^=0.01.

In the two group case, the data are generated from model (2), where *J*=1,2, and the number of observations is *K*_*i*1_=15 for the “wild” group and *K*_*i*2_=22 for the “mutant” group, *i*=1,…,*n*. The sampling times are the same as those in the yeast cell cycle data, locating within interval [*a*_2_,*b*_2_]=[100,244]. For the true gene expression profiles, we consider model $X_{\text{ij}}(t)=\overset{2}{\underset{l=1}{\sum }}(\xi_{\text{ijl}}+\gamma_{\text{ijl}})\psi_{l}(t)$, where $\psi_{1}(t)=-\sqrt{2/(b_{2}-a_{2})}\cos\left(\right)2\Pi (t-a_{2})/(b_{2}-a_{2})))$, $\psi_{2}(t)=\sqrt{2/(b_{2}-a_{2})}\sin\left(\right)2\Pi (t-a_{2})/(b_{2}-a_{2})))$, *t*∈[*a*_2_,*b*_2_]. The coefficients $\xi_{\text{ijl}}∼\mathrm{i.i.d.}\;N(0,\lambda_{\mathrm{\xi l}})$, $\gamma_{\text{ijl}}∼\mathrm{i.i.d.}\;N(0,\lambda_{\mathrm{\gamma l}})$, with (*λ*_*ξ*1_,*λ*_*ξ*2_)=(4,2) and (*λ*_*γ*1_,*λ*_*γ*2_)=(5,3). For non-differentially expressed genes, we let *γ*_*ijl*_=0. The error term is generated from N(0,0.01).

For both cases, We generate *n*=1000 genes and the proportion of differential genes is set to be *Π*_0_=0.05,0.2,0.5, respectively. The number of principal components in our method is selected so that the fraction of variation explained (FVE) exceeds 90%. This criterion selects the correct number of components for over 90% of time under all simulation scenarios. We tried the method proposed by [12], which fits all the top “eigen-genes”, to select the number of B-spline basis for EDGE and Sohn’s method. For over 80% of time, we ended up with selecting 19 basis for the single group case and 13 basis for the two group case, leading to severely under-smoothed gene expression curves and very few differential genes identified. We therefore manually select 6 bases for the single group case and 5 bases for the two group case for EDGE and Sohn’s methods, which seem to provide the best results when experimenting from 5 bases to 10 bases.

We perform 100 simulations for each simulation setting and the results are summarized in Tables 3 and 4. We compare the performance of our FPCA method in detecting differentially expressed genes with that of the EDGE and Sohn’s methods, based on the empirical false positive rate (FPR, proportion of the falsely rejected hypotheses over the total number of rejected hypotheses) and senstivity (proportion of true positives correctly identified). In our simulation studies, we also evaluated the performance of p-values calculated by (7), ‘Proposed^*b*^’ in Tables 3 and 4, and by (8), ‘Proposed^*a*^’ in Tables 3 and 4. We find that it is better to use (8) for computing the *p*-values when the proportion of differential genes is small, and throughout all settings, using (8) provides smaller false positive rates. We also find that under all scenarios, the proposed method clearly outperforms the EDGE method and Sohn’s method, especially when the proportion of differential genes is small. The fact that our method has the highest sensitivity is in line with the finding that our method identified the most genes in the application of yeast cell cycle data.

**Table 3:** Comparison of the proposed method, EDGE and Sohn’s method for the single group test by FPR (proportion of falsely rejected hypotheses over the total number of rejected hypotheses) and sensitivity (proportion of true positives correctly identified)

			**Proposed**^***a***^	**Proposed**^***b***^	**EDGE**	**Sohn’s**
*Π*_0_=0.05	FDR = 0.01	FPR	0.0016	0.0127	0.0100	0.0091
		Sensitivity	0.7606	0.6864	0.4742	0.5160
	FDR = 0.05	FPR	0.0180	0.0541	0.0459	0.0514
		Sensitivity	0.8440	0.7858	0.6378	0.6616
*Π*_0_=0.2	FDR = 0.01	FPR	0.0003	0.0084	0.0079	0.0078
		Sensitivity	0.7716	0.7851	0.6210	0.6302
	FDR = 0.05	FPR	0.0070	0.0409	0.0363	0.0408
		Sensitivity	0.8644	0.8667	0.7652	0.7641
*Π*_0_=0.5	FDR = 0.01	FPR	0.0002	0.0053	0.0041	0.0051
		Sensitivity	0.7731	0.8329	0.7021	0.7031
	FDR = 0.05	FPR	0.0025	0.0251	0.0238	0.0244
		Sensitivity	0.8709	0.9049	0.8285	0.8261

Proposed ^***b***^ uses (7) for computing *p*-values and Proposed ^***a***^, EDGE and Sohn’s use (8).

**Table 4:** Comparison of the proposed method, EDGE and Sohn’s method for the two group test by FPR (proportion of falsely rejected hypotheses over the total number of rejected hypotheses) and sensitivity (proportion of true positives correctly identified)

			**Proposed**^***a***^	**Proposed**^***b***^	**EDGE**	**Sohn’s**
*Π*_0_=0.05	FDR = 0.01	FPR	0.0071	0.0168	0.0079	0.0055
		Sensitivity	0.6186	0.5638	0.4858	0.4706
	FDR = 0.05	FPR	0.0432	0.0624	0.0478	0.0377
		Sensitivity	0.7048	0.6810	0.5850	0.5756
*Π*_0_=0.2	FDR = 0.01	FPR	0.0028	0.0099	0.0076	0.0032
		Sensitivity	0.6530	0.6566	0.5819	0.5153
	FDR = 0.05	FPR	0.0276	0.0440	0.0400	0.0200
		Sensitivity	0.7538	0.7579	0.6888	0.6505
*Π*_0_=0.5	FDR = 0.01	FPR	0.0013	0.0062	0.0047	0.0011
		Sensitivity	0.6701	0.7127	0.6370	0.5364
	FDR = 0.05	FPR	0.0134	0.0286	0.0238	0.0092
		Sensitivity	0.7850	0.8089	0.7465	0.6817

Proposed^***b***^ uses (7) for computing *p*-values and Proposed ^***a***^, EDGE and Sohn’s use (8).

## Conclusions

We proposed a new method for significance analysis of time course gene expression data by integrating a functional principal component method into a hypothesis testing framework. Our method can be applied to both single group and multiple group scenarios, and has shown to be more powerful in identifying temporally differentially expressed genes than the existing methods through real data application and various simulation studies. Moreover, our method is generally applicable, no matter the time course expression data have replicates or not, while most of the existing methods require replicates or even longitudinal replicates.

FPCA is a flexible nonparametric method for analyzing continuous trajectory data. The time course data are modeled through a data-based eigen-representation and the eigen-basis functions reflect the major modes of variation in the data. As illustrated in the yeast cell cycle data, the eigenfunctions often have a direct biological interpretation and offer a visual tool to assess the main directions in which the gene expression profiles vary. In addition, these eigenfunctions are orthogonal basis, so they carry information in a most efficient way and the representation of temporal trajectories can be more parsimonious than using the predetermined basis. This is particularly important in the significance analysis of time course gene expression data, because we could reserve more degrees of freedom for the inference.

In the EDGE and Sohn’s methods, the test statistic is constructed as a goodness-of-fit measure for each gene separately. Although our method uses a similar statistic, part of our statistic involves information from all genes, as the gene-specific expression curve and the variance stabilizer *δ* in the *F*-statistic (6) are estimated by borrowing strength from all the genes. This strategy can improve the power of the inference, especially for short time course data or data without replicates. In a simulation for one group test with only 11 measurements and no replicates for each gene (results not shown), our method can identify about 50% of differential genes correctly for an FDR of 0.01 and about 72% for an FDR of 0.05, but the EDGE and Sohn’s methods can hardly identify any differential genes.

Our method is also computationally fast. For the yeast cell cycle data, on a dual core processor 2.99GHz PC with 1.95 GB RAM, it took 385 seconds for the proposed method, 885 seconds for EDGE and 493 seconds for Sohn’s method to complete the one group test with *n*=10928 genes and *B*=10000 permutations/bootstraps, and 51, 176 and 189 seconds, respectively, for the two group test with *n*=1275 and *B*=10000. This ensures that our method can be applied to analyze very large genome wide data sets.

In our method, the covariance function is assumed to be the same for all genes in the same experimental group. This strategy has also been adopted by many other works in the analysis of time course gene expression data, for example [13] and [15]. A similar assumption was adopted in [6], where the within-gene correlation is implied in the presence of first-order dependence structure of the underlying Markov process and it is assumed to be identical for all genes. Although our method is presented assuming a homogeneous covariance, it can be easily extended to accommodate the heterogeneity in the covariance of gene expressions. We can first cluster the data and compute the covariance of gene expressions for each cluster, and then combine them to obtain the covariance of the mixed population [15].

When smoothing the covariance function, the bandwidth is chosen by generalized cross-validation (GCV). The overall shapes of the estimated covariance and eigenfunctions are quite stable over a range of bandwidth values in our numerical examples. The smoothing parameter may have effects on the power of the inference procedure, but the detailed investigation on this problem is beyond the scope of this paper. Intersected minds are referred to [27] and references therein for further discussions. Another related topic is to incorporate the inter-gene correlations in the multiple testing procedure, which has been discussed in [28,29] and [30]. Our method can be applied in combination with any of these multiple testing adjustment methods. However, the effect of different multiple testing adjustments on the results of significant testing is not the emphasis of this paper and could be an interesting topic for future research.

## Competing interests

The authors declare that they have no competing interests.

## Authors’ contributions

SW proposed the method, performed the statistical analysis and wrote the manuscript. HW supported the research project and revised the manuscript critically for important intellectual content. All authors read and approved the final manuscript.
